# Patterns of Evolution in the Unique tRNA Gene Arrays of the Genus *Entamoeba*

**DOI:** 10.1093/molbev/msm238

**Published:** 2007-11-01

**Authors:** Blessing Tawari, Ibne Karim M. Ali, Claire Scott, Michael A. Quail, Matthew Berriman, Neil Hall, C. Graham Clark

**Affiliations:** * Department of Infectious and Tropical Diseases, London School of Hygiene and Tropical Medicine, London, United Kingdom; † The Sanger Institute, Wellcome Trust Genome Campus, Hinxton, Cambridge, United Kingdom; ‡ The Institute for Genomic Research, 9712 Medical Center Drive, Rockville, Maryland

**Keywords:** *Entamoeba*, tRNA genes, repeated DNA, recombination

## Abstract

Genome sequencing of the protistan parasite *Entamoeba histolytica* HM-1:IMSS revealed that almost all the tRNA genes are organized into tandem arrays that make up over 10% of the genome. The 25 distinct array units contain up to 5 tRNA genes each and some also encode the 5S RNA. Between adjacent genes in array units are complex short tandem repeats (STRs) resembling microsatellites. To investigate the origins and evolution of this unique gene organization, we have undertaken a genome survey to determine the array unit organization in 4 other species of *Entamoeba—Entamoeba dispar*, *Entamoeba moshkovskii*, *Entamoeba terrapinae*, and *Entamoeba invadens*—and have explored the STR structure in other isolates of *E. histolytica*. The genome surveys revealed that *E. dispar* has the same array unit organization as *E. histolytica*, including the presence and numerical variation of STRs between adjacent genes. However, the individual repeat sequences are completely different to those in *E. histolytica*. All other species of *Entamoeba* studied also have tandem arrays of clustered tRNA genes, but the gene composition of the array units often differs from that in *E. histolytica/E. dispar*. None of the other species' arrays exhibit the complex STRs between adjacent genes although simple tandem duplications are occasionally seen. The degree of similarity in organization reflects the phylogenetic relationships among the species studied. Within individual isolates of *E. histolytica* most copies of the array unit are uniform in sequence with only minor variation in the number and organization of the STRs. Between isolates, however, substantial differences in STR number and organization can exist although the individual repeat sequences tend to be conserved. The origin of this unique gene organization in the genus *Entamoeba* clearly predates the common ancestor of the species investigated to date and their function remains unclear.

## Introduction

In 2005, *Entamoeba histolytica* HM-1:IMSS became the first Amoebozoan with a published genome sequence (Loftus et al. 2005). Although our understanding of its chromosome organization is still somewhat incomplete, a number of striking findings were made. Among these was the recognition that the tRNA genes were exceptionally abundant, with an estimated 4,500 copies—about 10 times the number in the human genome (Clark, Ali, et al. 2006). Additionally, most are clustered and organized into long tandem arrays that make up over 10% of the genome. Twenty-five distinct arrays have been described and are composed of tandemly repeated units encoding between 1 and 5 tRNA acceptor types. Three arrays also encode the organism's 5S RNA and 1 encodes another RNA suspected to be a small nuclear RNA (snRNA).

The intergenic regions between all arrayed tRNA genes in *E. histolytica* contain complex structures made up of short sequences repeated in tandem. These repeats are of varying size but generally fall in the range of 7–12 bp, although a few are substantially longer (up to 44 bp). In many ways the structures resemble the micro- and minisatellites seen in other eukaryotic genomes. However, micro- and minisatellites are normally randomly dispersed throughout the genome, whereas the tRNA-linked *E. histolytica* short tandem repeats (STRs) form part of a larger unit that is itself tandemly arrayed. Because *E. histolytica* appears to lack “traditional” mini/microsatellites elsewhere in its genome, we have chosen to use the term STR for these structures in *E. histolytica*, to emphasize the apparent distinction between these loci and the mini/microsatellites of other organisms.

Although clustering of an organism's tRNA genes has occasionally been observed in other eukaryotes, tandem arrays of tRNA genes have been reported to date only in *E. histolytica*. In the most closely related organism for which a genome sequence is available, *Dictyostelium discoideum*, individual tRNA genes are distributed throughout the genome (Eichinger et al. 2005). Observation of a unique gene organization inevitably raises the question of its origins, evolution, and function. To start to address these questions, we have investigated the organization of tRNA genes in other species of *Entamoeba* and other isolates of *E. histolytica*. For 4 species, a genome survey was performed, with 20,000 random sequences being obtained for each. The survey sequences have allowed an essentially complete picture of tRNA gene organization to be deduced for each of the 4 species and comparisons have yielded important insights into tRNA array evolution. Sequencing of the intergenic regions from dozens of isolates of *E. histolytica* has clarified the patterns of tRNA-linked STR evolution in that species.

## Materials and Methods

### DNA Isolation

*Entamoeba dispar* SAW760, *Entamoeba moshkovskii* FIC and Laredo, *Entamoeba terrapinae* M, *Entamoeba invadens* IP-1, and *Entamoeba ecuadoriensis* EC were originally obtained from the American Type Culture Collection (accession numbers 50484, 30041, 30042, 30043, 30994, and 50261, respectively). Organisms were grown in LYI-S-2 medium (Clark and Diamond 2002) with 15% (*E. dispar* and *E. ecuadoriensis*) or 10% (others) heat-inactivated adult bovine serum at 36 °C (*E. dispar* and *E. ecuadoriensis*) or 22 °C (others). *Entamoeba ecuadoriensis* cultures were supplemented with antibiotic-inhibited *Escherichia coli* XL-1 cells; the other species were grown axenically. Isolation of *Entamoeba equi* Aberystwyth DNA was described previously (Clark, Kaffashian, et al. 2006), whereas *E. moshkovskii* GAH and WS genomic DNAs were a gift from Jeffrey D. Silberman, University of Arkansas.

DNA samples from *E. histolytica* isolates for STR comparison were obtained during development of a genotyping method (Ali et al. 2005), a majority being from Bangladesh with the others coming from 11 different countries.

### Genome Survey Sequencing

The genomes of *E. dispar* SAW760, *E. moshkovskii* FIC, *E. terrapinae* M, and *E. invadens* IP-1 were surveyed by sequencing of small insert libraries (ca. 2.5 kb). For genomic library construction, axenic cells were harvested, washed, embedded in low–melting point agarose (CleanCut agarose, Bio-Rad Laboratories Ltd, Hemel Hempstead, UK) and cast into pulsed field gel electrophoresis blocks. Blocks were incubated in lysis buffer with proteinase K at 55 °C for 2 days. After electrophoresis, high molecular weight DNA was purified by agarase digestion, sonicated, and cloned into pUC18 before sequencing. More detailed information on library construction can be found at: http://www.sanger.ac.uk/Teams/Team53/psub/methods.shtml. Based on the average read length (ca. 600 bp) of the 20,000 reads and assuming a similar genome size to *E. histolytica* (ca. 23 Mb) in all species, the genome coverage of the surveys is approximately 0.5×.

### Other Sequencing

For routine analyses, DNA was isolated, polymerase chain reaction (PCR) amplification performed, and PCR products separated in 1.2–1.5% agarose gels as previously described (Ali et al. 2005). Products were purified using the QiaQuick gel extraction kit (Qiagen Ltd., Crawley, UK), sequenced directly (BigDye v.3.0, Applied Biosystems, Warrington, UK) using the individual amplification primers, and analyzed on an ABI3730 sequencer.

For *E. ecuadoriensis*, *E. moshkovskii* Laredo, and *E. equi*, individual array units were obtained by PCR amplification using tRNA primers (Ali et al. 2005), gel purified as above, cloned into pGEM-T Easy (Promega UK, Southampton, UK), and individual plasmids sequenced using M13F and R primers. A few gaps in sequences from the genome surveys were also filled by this method. *E. moshkovskii* GAH and WS PCR products were sequenced directly using amplification primers.

### Sequences Deposited

The genome survey read data have been deposited in the EMBL database with the following accession numbers: *E. invadens* AM600998–AM623528, *E. dispar* AM623529–AM643666, *E. moshkovskii* AM643667–AM665026, and *E. terrapinae* AM665027–AM689296. Consensus sequences of tRNA array units from the 4 genome surveys, and other species, and representative sequences from the *E. histolytica* intraspecific diversity study have been deposited in the databases under the accession numbers EF421258–EF421403 and EF427346–EF427363.

### tRNA Gene Identification

Raw genome survey sequences were scanned using tRNAscan SE (Lowe and Eddy 1997). Reads encoding the same tRNA isoacceptor type were aligned (http://prodes.toulouse.inra.fr/multalin/multalin.html; Corpet 1988), manually edited, and a consensus sequence obtained.

### STR and Scaffold/Matrix Attachment Regions Identification

Tandem Repeat Finder (http://tandem.bu.edu/trf/trf.html; Benson 1999) was used to search for STRs in the consensus sequences. Scaffold/matrix attachment regions (S/MAR) were predicted using MARSCAN (http://bioweb.pasteur.fr/seqanal/interfaces/marscan.html).

### Nomenclature

Array unit names are derived from the encoded tRNAs (and 5S RNA, when present) using the standard single-letter amino acid abbreviation and are depicted in square brackets. The anticodon is included where necessary to distinguish between arrays. Regions of an array are also identified using the single-letter code and anticodons but without the square brackets. For example, A-S and S^GCT^-D are both intergenic regions in the *E. histolytica* array [ASD]. The anticodon is necessary for S^GCT^-D as another array, [SD], contains S^TGA^-D. STR loci are also named after the tRNA genes flanking the intergenic region in which they are found, for example, STR locus S^GCT^-D.

## Results

**Fig. 1:**
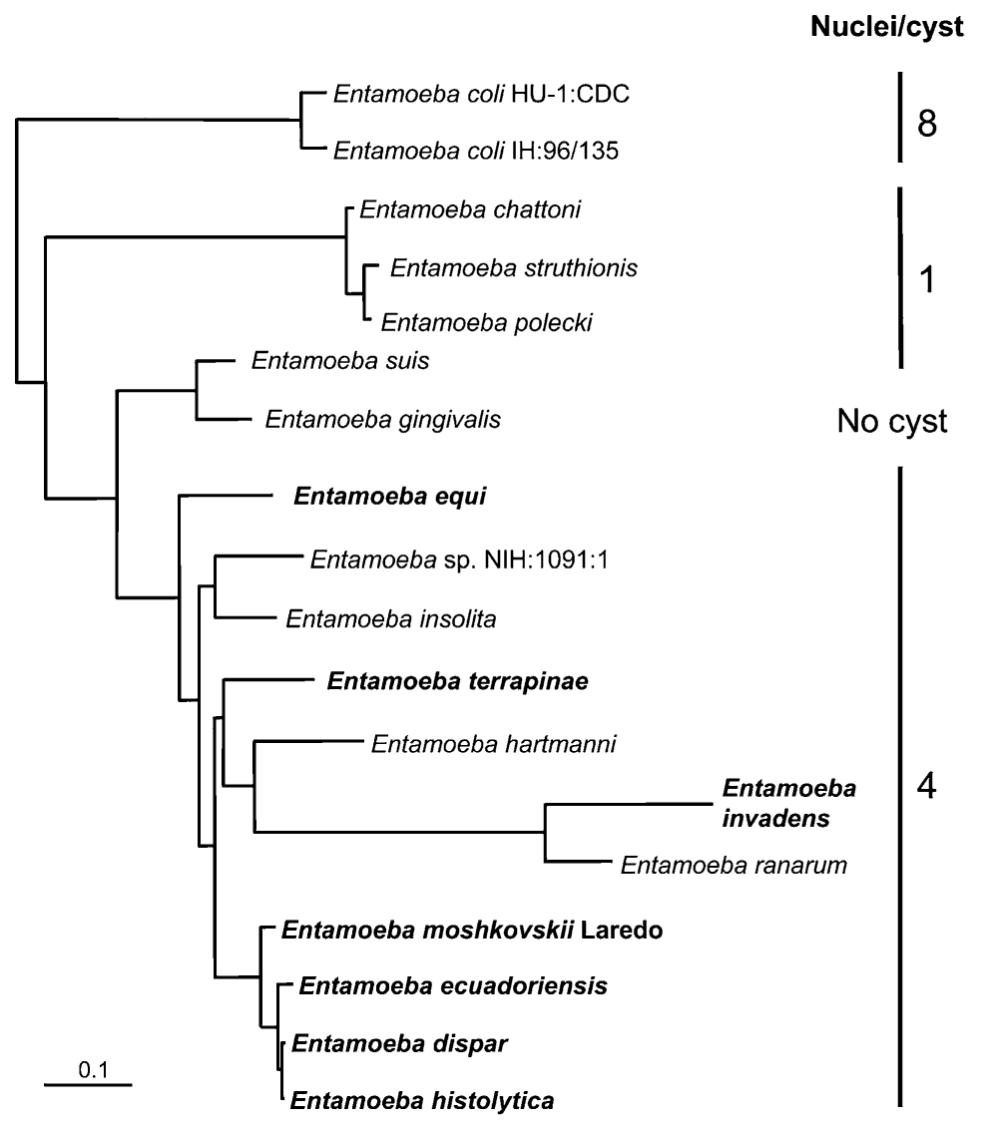
Phylogenetic relationships among *Entamoeba* species. The tree depicted is redrawn from that in Clark, Kaffashian, et al. (2006). Species producing cysts with different nuclear number are indicated. Species referred to in this work are shown in boldface. The scale bar represents the evolutionary distance equivalent to 0.1 changes per site.

The genus *Entamoeba* consists of species that produce cysts with 1, 4, or 8 nuclei and a few that do not encyst. At present, it appears that cyst nuclear number is a reasonably good indicator of phylogenetic relatedness, with all the 4-nucleated cyst producers forming a robust clade (Clark, Kaffashian, et al. 2006). To date, only species in the 4-nucleated cyst clade have been grown successfully in the absence of bacteria. The species chosen for our genome survey analysis cover the range of diversity among *Entamoeba* species that can be grown in axenic culture (fig. 1).

### *Entamoeba histolytica/E. dispar* comparisons

Phylogenetic analyses have consistently shown that *E. histolytica* and *E. dispar* are sibling species (Silberman et al. 1999; Clark, Kaffashian, et al. 2006). Indeed, it is only in the past 10–15 years that they have been widely accepted as being distinct (Diamond and Clark 1993). The assembly into arrays of tRNA gene-containing sequences from the *E. dispar* SAW760 genome survey allowed comparisons between these species to be performed. The gene content of the tRNA array units is identical in the 2 species. The only difference is that although most *E. histolytica* isolates (including the genome strain HM-1:IMSS) have 2 versions of the array containing Asn^GTT^ and Lys^CTT^ genes ([NK1] and [NK2]), which differ completely in their STR regions and other intergenic sequences (Ali et al. 2005), the *E. dispar* SAW760 genome contains only 1 type of [NK] array. Thus, *E. dispar* SAW760 has 24 arrays rather than the 25 found in *E. histolytica* HM-1:IMSS. Note, however, that a few *E. histolytica* isolates have lost 1 array ([NK1]) and therefore have 24 arrays (Ali et al. 2005). It is possible that the single [NK] array in *E. dispar* SAW760 may also prove to be an exception.

The tRNA/5S RNA gene content and orientation of all arrays is identical in the 2 species. However, the corresponding intergenic sequences generally share no similarity in either their STR or simple sequence regions. This contrasts dramatically with *E. histolytica* intraspecific variation where, despite substantial STR number variation, the simple sequence regions and the individual repeat sequences that make up the STRs are highly conserved (see below). The ability to design species-specific primers for amplification of intergenic regions from all isolates of each species also confirms that the sequences immediately adjacent to the genes are conserved within each species (Zaki et al. 2002; Ali et al. 2005).

### *Entamoeba moshkovskii* Organization

Phylogenetic analyses have consistently shown that *E. moshkovskii* is closely related to *E. histolytica* and *E. dispar* (Silberman et al. 1999; Clark, Kaffashian et al. 2006). The organisms are morphologically identical and some *E. moshkovskii* subtypes have been shown to infect humans, causing a diagnostic problem (Ali et al. 2003). It was not surprising, therefore, to find that most arrays in *E. moshkovskii* FIC show the same gene content and orientation as in *E. histolytica* and *E. dispar*. Indeed, of the 23 arrays in *E. moshkovskii* 19 have the same organization as arrays in *E. histolytica* and *E. dispar* (table 1). There is one major difference, however. In no case is there any evidence for STRs in the intergenic regions. On average the *E. moshkovskii* array units are significantly smaller than their homologs in *E. histolytica* and *E. dispar* (mean size 64% and 61%, respectively).

### *Entamoeba moshkovskii* Isolate Differences

To further investigate the differences in array unit organization between *E. moshkovskii* and *E. histolytica/E. dispar*, a few units were sequenced in other *E. moshkovskii* isolates including some that belong to a different subtype. Again, no STRs were observed; nevertheless, differences in the intergenic region were substantial, both in sequence and length (data not shown). For intergenic region W-I, 2 distinct unit variants were observed in both *E. moshkovskii* FIC and *E. moshkovskii* Laredo and the relative similarity suggests that the divergence between the unit variants postdated divergence of the *E. moshkovskii* strains. The intergenic regions are much more similar within than between strains, although both length and sequence differences are still present. This suggests either that duplication and divergence of arrays occurred independently, or that some “cross-talk” between the 2 arrays has occurred subsequent to strain divergence. There is no evidence of repeats of any type being responsible for the length differences observed in any of the intergenic regions for which sequences from multiple isolates are available.

### *Entamoeba terrapinae* and *E. invadens*

These 2 reptilian parasites are not closely related to each other or to the 3 species already mentioned. Not surprisingly, their array organizations are also very different, with only 8 of the 26 distinct arrays in *E. terrapinae* M and 4 of the 20 arrays in *E. invadens* IP-1 having the same gene organization as in *E. histolytica* (table 1). Among the *E. invadens* arrays is the largest one seen to date with a unit size of 2364 bp, containing 6 tRNA genes and a 5S RNA gene (array [VMEDR5E]). One peculiarity of *E. invadens* is the existence of an array containing only 5S RNA genes. This is the arrangement of 5S RNA genes found in many other eukaryotes, including humans, but it is not present in the other *Entamoeba* species studied to date.

There is no evidence for non-tRNA/5S RNA genes being part of arrays in the surveyed species, except for homologs of the gene encoding small RNA “X” previously identified in an *E. histolytica* array (Banerjee and Lohia 2003; Clark, Ali, et al. 2006). This ~200-nt RNA is thought perhaps to be a small nuclear RNA and is always encoded in arrays adjacent to the gene for tRNA Thr^TGT^. It is notable that snRNAs have been found occasionally encoded in arrays with the 5S RNA genes of a few organisms, including certain fish (Manchado et al. 2006) and oysters (Cross and Rebordinos 2005). In the latter case there is also a microsatellite present.

### Cross-Species Comparison of Array Organization

**Fig. 2:**
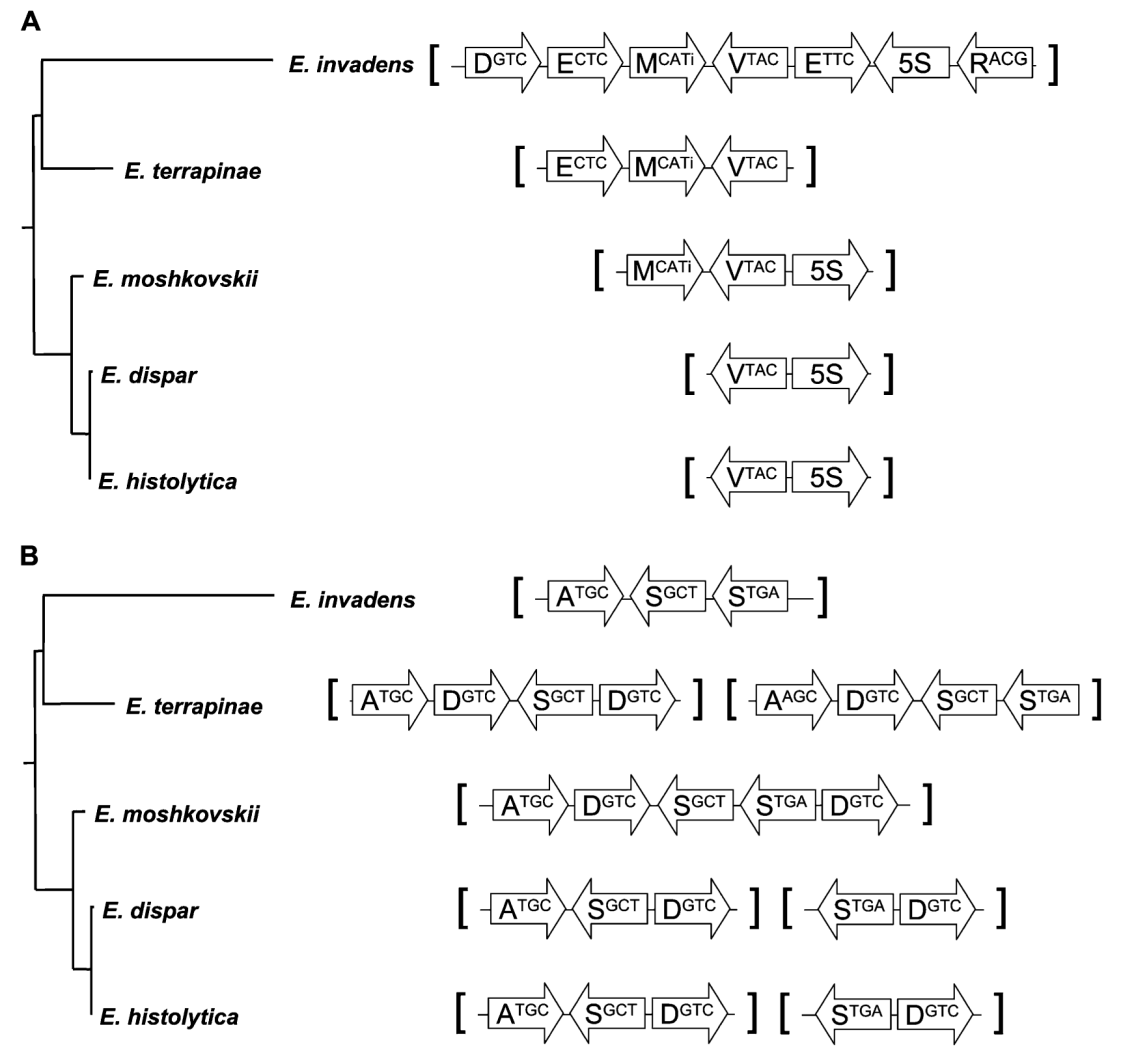
Mapping of array organization onto phylogenetic tree. The relationships among surveyed species from figure 1 (*Entamoeba invadens* branch shortened for simplicity) are shown adjacent to a depiction of the corresponding array. Arrows indicate the orientation of the tRNA/5S RNA gene and contain the single-letter amino acid code and anticodon for the encoded tRNA. (*A*) The array unit organization involving the gene encoding tRNA Val^TAC^. (*B*) The array unit organization involving the genes encoding tRNAs Ser^GCT^ and Ser^TGA^.

**Fig. 3:**
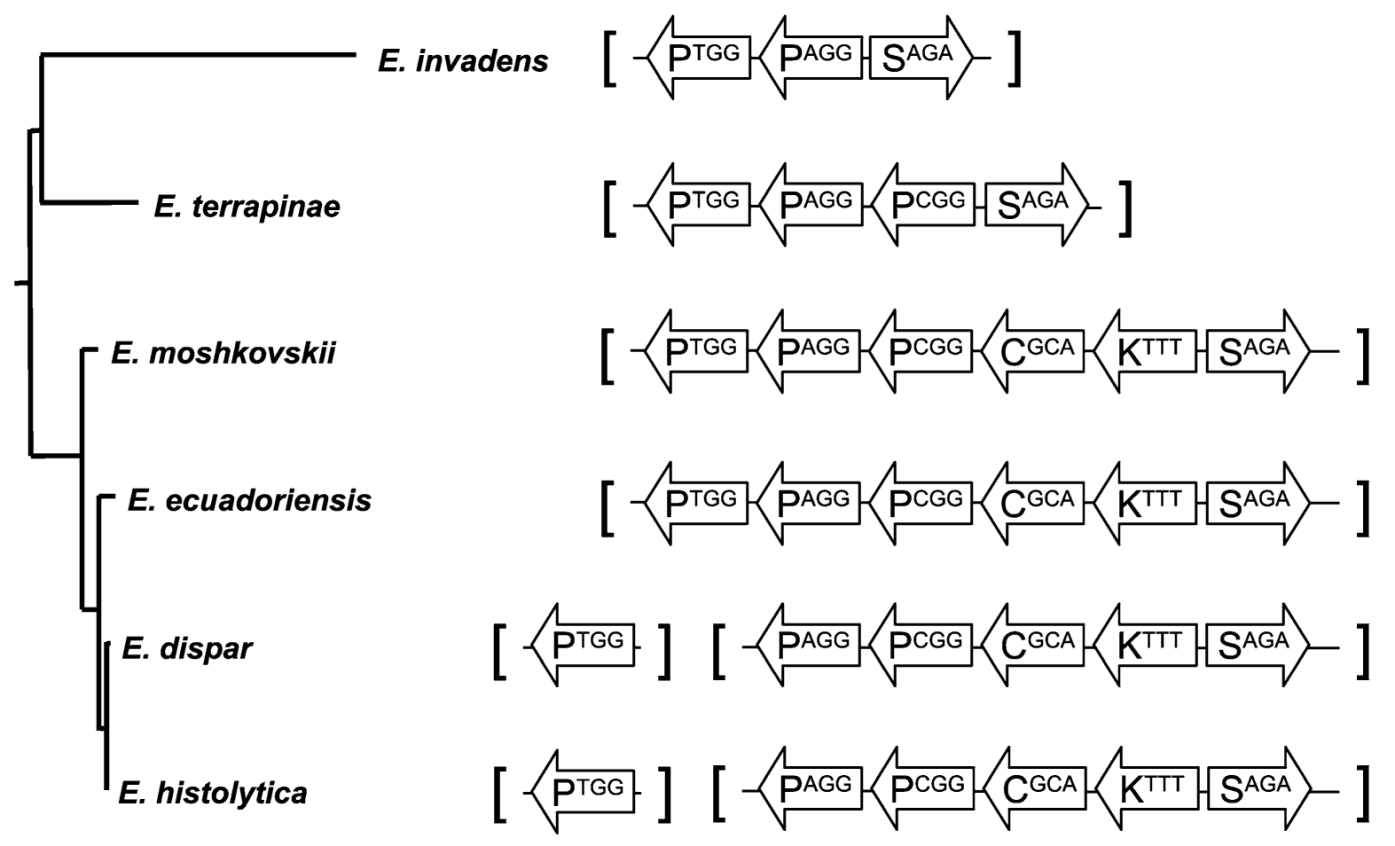
Array organization of genes encoding Pro^TGG^. The relationships among surveyed species plus *Entamoeba ecuadoriensis* from figure 1 (*Entamoeba invadens* branch shortened for simplicity) are shown adjacent to a depiction of the corresponding tRNA-Pro-encoding arrays. Arrows indicate the orientation of the tRNA gene and contain the single-letter amino acid code and anticodon for the encoded tRNA.

Despite the small number of array units with identical organization between the reptilian species and *E. histolytica/E. dispar*, it is nevertheless easy to see similarities—the context and orientation of adjacent tRNA genes is frequently conserved even if the overall unit organization differs (figs 2 and 3). Indeed, it is possible to produce a network of array structures in which similar tRNA gene arrangements are linked (supplementary figs. 1–5, Supplementary Material online). In several cases (e.g., Val^TAC^ in fig. 2A and Ser^GCT^ in fig. *2B*), the gene is in a distinct gene/array combination in each species (except *E. histolytica/E. dispar*) but the similarities in context are nevertheless clear. In most cases, the direction of the fission/fusion events cannot be stated with any certainty, but in 2 cases the order of events can be deduced.

In *E. histolytica/E. dispar*, tRNA Pro^TGG^ is encoded on its own in a single-gene array, whereas in the other species it is part of a larger unit and adjacent to a gene encoding tRNA Pro^AGG^ (fig. 3). The species most closely related to *E. histolytica/E. dispar* is the little-studied organism *E. ecuadoriensis* EC. The equivalent array was cloned and sequenced from this species and its organization shown to be identical to that in *E. moshkovskii*, namely [SPPPCK]. The most parsimonious explanation for this distribution of organizations is that in the *E. histolytica/E. dispar* lineage, the tRNA Pro^TGG^ gene has “popped out” of the [SPPPCK] array that was present in the common ancestor they shared with *E. ecuadoriensis* to form a separate array on its own (fig. 3). Similarly, *E. ecuadoriensis* has an Asp^GTC^ gene between Ala^TGC^ and Ser^GCT^ genes, as in *E. moshkovskii* (fig. 2B), again suggesting that fission of the [ADSSD] unit into [ASD] and [SD] occurred in the *E. histolytica/E. dispar* lineage after divergence from *E. ecuadoriensis*.

### Dispersed tRNA Genes

In *E. histolytica*, 5 tRNA isoacceptor types were found to be encoded by nonarrayed genes, dispersed in small numbers throughout the genome. Most of the dispersed tRNA genes found in the other *Entamoeba* genomes encode the same tRNAs as in *E. histolytica*. However, there are some exceptions: tRNA Thr^CGT^ is encoded as part of an array with tRNA Gln^TTG^ in all species except *E. terrapinae*, and all species have a [Gly^TCC^] array except for *E. invadens*, where it is dispersed (table 1). Additionally, there are 2 types of tRNA Leu^TAA^ gene that are distinct in sequence, one of which has an intron (Leu^TAAi^). In *E. histolytica/E. dispar* and in *E. moshkovskii*, the gene with the intron is dispersed and the other gene is arrayed. Both types of gene are also present in *E. terrapinae* and *E. invadens*, where they are both found in arrays (table 1).

### Cross-Species Comparison of Array Base Composition

In the analysis of *E. histolytica* arrays (Clark, Ali, et al. 2006), it was noted that the regions of the arrays units between genes were A + T rich (on average 80.0%) and showed a pyrimidine/purine compositional bias (on average 70.8% pyrimidine on one strand). The arrays for the 4 new species were similarly analyzed (table 2). All intergenic regions were A + T rich, although those in *E. moshkovskii* and *E. terrapinae* were 10% lower in A + T than the other species. The pyrimidine/purine compositional bias was also strong in *E. dispar* and *E. moshkovskii* but was much less pronounced in *E. invadens* and essentially absent in *E. terrapinae* arrays. Although this bias is likely to affect DNA structure when present, the lack of conservation of this feature suggests that it is not essential to the present role of the arrays across all species.

### *Entamoeba histolytica* Intraspecific STR Variation

During the development of a genotyping method (Zaki and Clark 2001; Ali et al. 2005), limited sequencing revealed that the basis of PCR product length variation between isolates was variation in the numbers of repeats in the inter-tRNA gene spacer regions. To investigate the patterns of variation, 3 of the STR loci used for genotyping were selected for study in greater depth: R-R from array [R^TCT^], S^TGA^-D from array [SD], and N-K2 from array [NK2]. In almost every case, an unambiguous sequence was obtained from direct sequencing of the PCR products, indicating that the majority of copies in each array share the same sequence. Nevertheless, it must be remembered that each sequence is a “consensus.”

Each of the 3 STR loci studied contains blocks of repeats that show both copy number and sequence variation. Within each block, there are often repeat sequence variants that differ by only 1 base. The start point of the repeat sequence is defined somewhat arbitrarily as “partial repeats” may also be present at the ends of each block.

### STR locus R-R

**Fig. 4:**
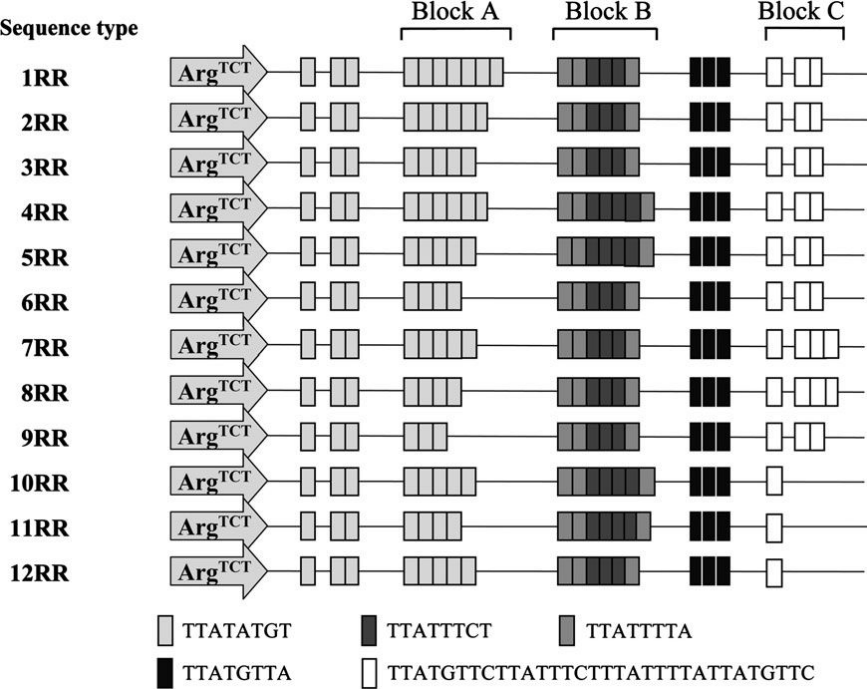
Intraspecific differences in STR organization in the *Entamoeba histolytica* R-R intergenic region. Sequence types identified through sequencing of STR regions from different isolates are shown. Blocks of STRs are indicated, with distinct repeat sequences being assigned different shading. The arrow indicates the position of the tRNA gene and contain the 3-letter amino acid code and anticodon for the encoded tRNA. The complete [R^TCT^] array unit is depicted as there is only 1 tRNA gene in this array.

The intergenic region in array [R^TCT^] contains 3 blocks with variable repeat copy numbers (Blocks A–C; fig. 4). Block A contains between 3 and 7 tandem copies of a single 8-base sequence. Adjacent to Block A is a tandem duplication of the same sequence that shows no copy number variation between isolates. Block B consists of two 8-base repeat sequence variants, one type occurring only at each end of the block, whereas the central variant can be present in either 3 or 4 copies. Three tandem copies of an 8-base sequence are always found between Blocks B and C. The 8-base repeats present in Blocks A and B and between Blocks B and C are all related in sequence.

Block C contains one of the largest repeats found in *E. histolytica*, a 32-base sequence. This repeat may be present as a single copy plus 2 or 3 tandem copies or the tandem copies may be missing completely, apparently having been deleted in some isolates. The sequence of the 32-base Block C repeat clearly consists of four 8-base sequences related to those in the other blocks. However, the nature of the interstrain variation shows that it evolves as a 32-base sequence.

In total, 12 different combinations of sequences for the 3 blocks have been identified in the [R^TCT^] intergenic region among the 136 sequences obtained to date. Of particular note is the fact that 3 pairs of sequence types have the same overall length (1RR/4RR, 2RR/5RR, and 11RR/12RR), resulting from compensating gains and losses of single repeats of 8 bases in Blocks A and B. This means that some PCR product sizes used in isolate identification can be derived from more than one sequence type.

### STR Locus S^TGA^-D

**Fig. 5:**
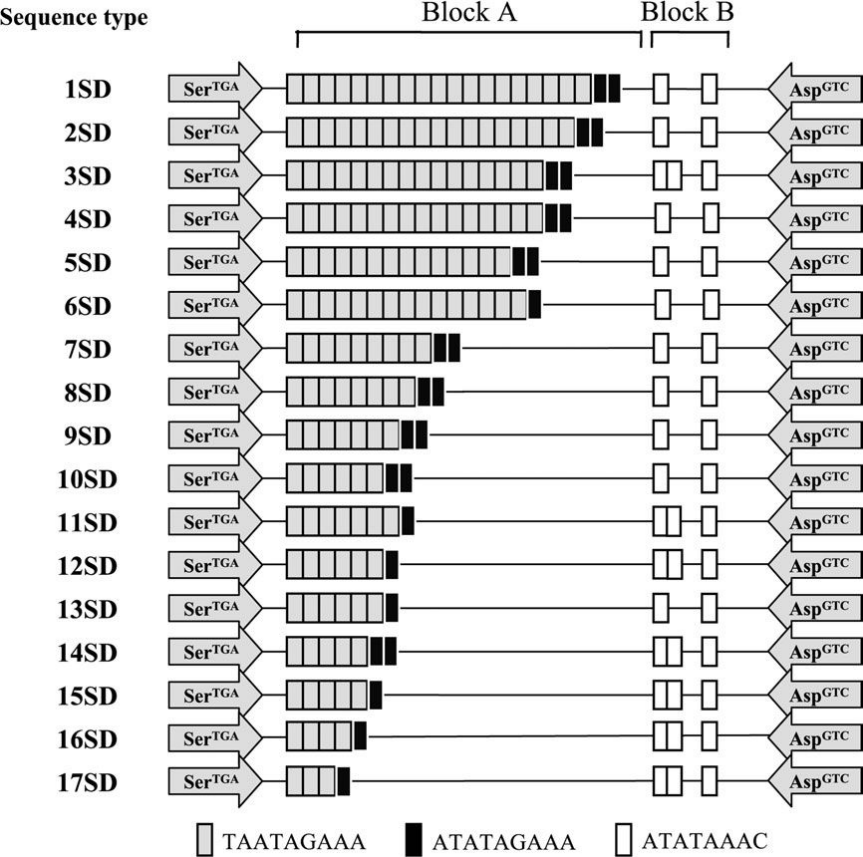
Intraspecific differences in STR organization in the *Entamoeba histolytica* S^TGA^-D intergenic region. Sequence types identified through sequencing of STR regions from different isolates are shown. Blocks of STRs are indicated, with distinct repeat sequences being assigned different shading. The arrows indicate the position of the tRNA genes and contain the 3-letter amino acid code and anticodon for the encoded tRNA.

This intergenic region shows 2 variable blocks (fig. 5). Block B consists simply of an eight-base sequence that can be present in 2 or 3 copies. In contrast, Block A shows extensive variation. Two 9-base repeat sequence variants that differ at one position make up Block A. The first sequence variant may be present in anything from 3 to 19 copies. The second variant is present in either 1 or 2 copies and always at the same end of Block A.

In total, 17 sequence types have been identified among the 128 intergenic regions sequenced to date, and once again certain distinct sequence types have essentially the same lengths as a result of compensating gains and losses of repeats (5SD/6SD, 9SD/11SD, 10SD/12SD/14SD, and 13SD/15SD).

### STR locus N-K2

**Fig. 6:**
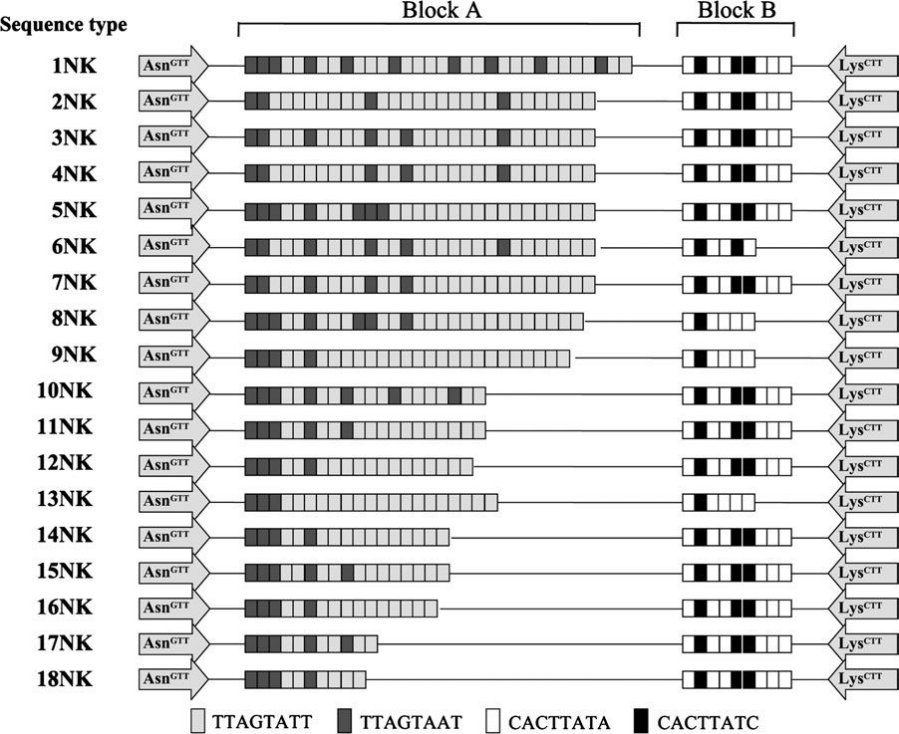
Intraspecific differences in STR organization in the *Entamoeba histolytica* N-K2 intergenic region. Sequence types identified through sequencing of STR regions from different isolates are shown. Blocks of STRs are indicated, with distinct repeat sequences being assigned different shading. The arrows indicate the position of the tRNA genes and contain the 3-letter amino acid code and anticodon for the encoded tRNA.

This intergenic region shows the greatest degree of diversity of the 3 for which substantial numbers of sequences are available, with 18 sequence types detected among the 53 sequences obtained (fig. 6). Again 2 blocks are present and each consists of two 8-base sequence variants that differ at one position. Although Block B is found in only 3 forms, Block A can contain from 10 to 32 copies of the repeats. Five of the sequence types are of the same length (2NK-5NK and 7NK) as are 2 other pairs (10NK/11NK and 14NK/15NK). In addition to copy number variation, different patterns of sequence-variant interspersion contribute to the diversity.

### Other Diversity

Most of the sequence diversity detected in the intergenic regions is confined to copy number variation in the repeat blocks. However, a number of point mutations have also been detected, primarily in the nonrepeated regions (data not shown). These are mostly transitions, but occasional transversions and indels have been noted. The mutations are scattered across the intergenic sequences and most have been seen in only 1 isolate. As well as point mutations observed when comparing sequences between isolates, sequencing of individual clones derived from the same PCR product also reveals point mutation differences between individual unit copies from the same array. That these are not amplification artifacts is confirmed by detection of similar features in clones sequenced during the genome project as these were derived from sheared genomic DNA rather than PCR products. Point mutations as well as occasional differences in STR number are observed in the individual reads, although they represent a small minority of the sequences in each case.

### Geographic Distribution

Eleven out of the 12 R-R sequence types were found among the 84 Bangladeshi samples studied, whereas 9 were found among the 54 samples from other countries. This suggests either that most sequence types have a wide geographic distribution or that they have arisen independently in multiple locations. A similar picture emerges from the other loci: 14 out of the 17 S-D sequence types were found in the 80 Bangladeshi samples, whereas 10 were found in 48 samples from elsewhere; 12 out of the 18 N-K2 sequence types were found in 36 Bangladeshi samples with 9 being found in the 18 samples from elsewhere in the world.

## Discussion

### Comparative Organization Versus Phylogeny

All of the *Entamoeba* species studied here belong to the 4-nucleated cyst-producing clade, and so at present we cannot say whether tRNA arrays are a characteristic of all species in the genus. Through the *E. histolytica* genome project and the genome surveys of 4 additional species reported here, we have a complete picture of the tRNA array organization in a cross section of species from this clade. It is possible that a few undetected single-copy dispersed tRNA or 5S RNA genes may exist in the surveyed species as the genome coverage in each case is low (ca. 0.5×). However, it seems unlikely that arrays will have been missed in the surveys and examples of all tRNA isoacceptor types detected in the *E. histolytica* genome have also been found in the other species.

In addition to the surveys, examples of tRNA array units from a few additional species have been sequenced. This was undertaken to clarify the patterns of evolution in the case of *E. ecuadoriensis* and to confirm the presence of arrays in the case of *E. equi*, the latter being the earliest branch of the 4-nucleated cyst clade in the most recent phylogenetic tree (Clark, Kaffashian, et al. 2006). Several array units have the same organization in all species examined, which suggests that these may be useful starting points for examining tRNA array organization in species outside this clade.

Only in *E. histolytica* and *E. dispar* are significant STRs observed in all array units. In other species, small numbers of tandem repeats are seen occasionally in the intergenic regions but there is no consistency to their presence. The one array unit sequenced in *E. equi*, [H^GTG^], exists in 2 variants and comes the closest to having a STR as there are 4 tandem copies of a 6-base sequence in each. In *E. ecuadoriensis*, 3 tandem copies of a 10-base sequence occur in array [SPPPCK], and in *E. invadens*, 3 tandem copies of an 8-base sequence occur in array [MR]. The other tandem repeats observed are very short (4 bases or less) and few in number or are simple duplications, probably arising by chance through mutation.

For 2 species, multiple isolates have been examined at several loci. It was already clear from limited sequencing that the main source of interisolate variation in *E. histolytica* tRNA arrays was variation in repeat number (Zaki and Clark 2001). The data presented here give an in-depth insight into 3 intergenic regions. However, they also raise new questions. It is unclear why different blocks of repeats in the same intergenic region show dramatically different patterns of diversity. For example, N-K2 has 2 blocks of repeats, 1 of which exists in 11 length variants and the other in only 2, despite the fact that the repeat size is the same and the blocks are separated by only 82 bases. Across all 3 intergenic regions for which significant data are available, there is no obvious pattern. The repeat size, base composition, and relative position in the array unit do not appear to determine whether a block is going to be variable or stable in repeat number.

In *E. moshkovskii*, a smaller number of isolates were compared. The underlying basis for the length variation observed is much less clear as no obvious repeat structure can be discerned in the intergenic regions of the array units studied. There is substantial sequence divergence between isolates. In part, this reflects the fact that *E. moshkovskii* is actually a species complex (Clark and Diamond 1997), but even between the sequences that can be aligned well the origins of the observed length differences remains unclear.

It is notable that substantial differences in tRNA gene number appear to exist between species. The percentage of reads encoding tRNAs varies over 10-fold, from 1.1% in *E. terrapinae* to 15.7% in *E. dispar*, indicating a range of between 500 and 7000 tRNA genes, assuming that no differences in “clonability” exist between arrays within or between species. That clonability is a potential confounding factor is illustrated by the observation that no sequences in the *E. terrapinae* survey contained His^GTG^ genes; that array sequence was obtained by direct sequencing of PCR products. If correct, the numbers imply significantly fewer tRNA genes and also shorter arrays in *E. moshkovskii*, as the average unit length differs less than 2-fold between *E. moshkovskii* and *E. dispar*. Nevertheless, even in *E. moshkovskii*, the total number of tRNA genes in the genome is likely to be substantially larger than in most eukaryotes.

### Origins

At present, we do not have data on the tRNA gene organization in subgroups of the genus *Entamoeba* other than the 4-nucleated cyst-producing clade, nor do we have data on the tRNA gene organization in subgroups of the genus *Entamoeba* other than the 4-nucleated cyst-producing clade nor do we have any indication of their organization in the most closely related genera identified to date (*Endolimax*, *Pelomyxa*, and *Mastigamoeba*; Silberman et al. 1999; Nikolaev et al. 2006). The origin of this unique gene organization thus cannot be deduced by comparison to related organisms.

The origin of the STRs within *E. histolytica* and *E. dispar* arrays may be less obscure if only by analogy to what has been observed in other systems. The inter-tRNA regions in these species are extremely A + T rich. As a result, short tandem “duplications” are likely to occur quite frequently by chance mutation. Strand slippage during DNA replication or repair then has the potential to result in repeat propagation (Ellegren 2004), resulting in the STR loci observed. Strand slippage is also likely to be the mechanism generating the interstrain diversity observed.

Of interest in this context is the observation that active tRNA genes appear to be “hot spots” for recombination, probably due to RNA polymerase III transcription complexes forming replication fork barriers, leading to replication pauses and resulting in “fragile” DNA (Deshpande and Newlon 1996; Labib and Hodgson 2007). This link of recombination to tRNA genes has been observed in both yeast (where it can result in chromosome rearrangements [Dunham et al. 2002; Admire et al. 2006; Pratt-Hyatt et al. 2006] and retrotransposon integration [Kim et al. 1998]) and *Dictyostelium* (where tRNA genes are closely linked to retrotransposon integration sites [Szafranski et al. 1999; Siol et al. 2006]). Retrotransposon sequences are frequently found in the regions immediately flanking *E. histolytica* tRNA arrays (Clark, Ali, et al. 2006) suggesting that a similar link exists here also. Pausing of replication may also increase the likelihood of strand slippage (Ellegren 2004), which is probably essential for tRNA array maintenance.

That *E. histolytica* (and presumably other species of *Entamoeba*) can undergo DNA repeat expansion was observed as long ago as 1992, when it was reported (Bhattacharya et al. 1992) that repeat numbers were increasing (and decreasing) in a region of the ribosomal DNA episomal circle over quite a short period of time in continuous culture. The repeats in that case were 170 bp in size, intermediate between the STR and tRNA array unit sizes. Whether comparable tRNA array unit copy number changes are occurring in culture is not known at this stage, but based on PCR product sizes, the average number of STRs in intergenic regions does not show rapid variation, the patterns observed in *E. histolytica* being stable across several years in continuous culture (Ali et al. 2005) and during axenization (Clark, unpublished data).

It is not difficult to envision how new tRNA genes can get incorporated into an existing array in a 2-step process. The new genes need to become linked to one unit in an array. This could occur by recombination among distinct array units or by insertion of reverse transcribed tRNAs (t-SINEs; Piskurek et al. 2003). The process responsible for homogenization of repeats in a tandem array is most likely concerted evolution. Concerted evolution has been most extensively studied in the rDNA of eukaryotes (Eickbush and Eickbush 2007) because these genes are usually (although not in *Entamoeba*) arranged in long tandem arrays as seen for the tRNA arrays described here. This gene conversion-based process will occasionally lead to the spread of that variant unit and the replacement of the existing arrayed copies. The spread of variants is a random process not requiring a selective advantage for the new unit structure. Other types of mechanism could also be involved, such as unequal sister chromatid exchange.

The reverse process is more difficult to envision, where one or more genes “leave” an existing array to form a separate array unit. This has occurred in 2 cases described here, the clearest being the Pro^TGG^ gene, which has popped out of array [SPPPCK] to form its own array in the *E. histolytica/E. dispar* lineage (fig. 3). Several ways in which this could have occurred can be envisioned. One or more Pro^TGG^ genes may have become “dispersed” from the array, before subsequently being eliminated from [SPPPCK]. The existence of dispersed copies would make elimination of the Pro^TGG^ gene from the array selectively neutral. A dispersed gene could then become rearrayed. Alternatively, 2 [SPPPCK] arrays may have coexisted in the genome and have undergone complementary gene eliminations, leading to [Pro^TGG^] in one case and [SPPCK] in the other—no dispersed genes are required in this version. The apparent secondary dispersal of the Leu^TAAi^ gene in the lineage leading to *E. histolytica*/*E. dispar*/*E. moshkovskii* may reflect the first stage of another such event occurring via the first scenario, although it is also possible that incorporation of this gene into arrays has occurred independently in *E. invadens* and *E. terrapinae*. Clustering of tRNA genes also occasionally occurs in the absence of the array organization, as 3 dispersed tRNA genes were found within a 0.6-kb region in *E. invadens* (#SGI in table 1). This may be indicative of the first stage in array formation.

### Function

The function of tRNA arrays in *Entamoeba* species remains obscure. The existence of low copy-number dispersed genes encoding certain tRNAs suggests that large gene numbers are not needed to provide sufficient tRNA for efficient translation, and there is no link between codon usage and tRNA gene number (Clark, Ali, et al. 2006). It is impossible to know whether all of the arrayed genes are transcribed, but there is no clear evidence from the surveys for any copies being nonfunctional pseudogenes, and it is difficult to envision a 100-fold difference in turnover rate between tRNA types. More likely is the possibility that many copies in an array exist in a transcriptionally inactive form due to differences in chromatin modification.

The first potential function to have been proposed is based on the observation of S/MAR consensus sequences in some of the *E. histolytica* array units (Banerjee and Lohia 2003). This consensus is a degenerate and A + T-rich sequence and was shown to occur frequently by chance in random sequences of the same base composition (Clark, Ali, et al. 2006). To further investigate this possible role, array units from *E. dispar* were analyzed using MARSCAN. Although S/MAR consensus sequences were found, they were frequently in different array units to those identified in *E. histolytica*. As the organization of the arrays is fully conserved in the 2 species, the lack of S/MAR conservation casts doubt on there being a matrix-binding role for the arrays.

A more recent proposal that the tRNA arrays may be located at chromosome ends and represent functional replacements for more traditional telomere repeats cannot yet be tested (Clark, Ali, et al. 2006). Assembly of the *E. histolytica* genome is not yet complete (Loftus et al. 2005), although efforts to achieve this are ongoing, and the coverage of the other genomes is too low to permit any testing of this theory. No telomerase or telomere-like sequences were found in the *E. histolytica* genome nor are they present in the genome surveys. Because *E. histolytica* chromosomes do not condense, cytogenetic localization of tRNA arrays to chromosome ends will not be possible. If the tRNA arrays are indeed telomeric, they face the same challenge as the structures they replace—how to replicate the ends without constant array length reduction.

## Conclusions

tRNA arrays appear to be a general feature of *Entamoeba* species' genomes, but the highly polymorphic STRs are largely confined to *E. histolytica* and *E. dispar*. The degree of similarity in organization reflects the phylogenetic relatedness of the species as indicated by small subunit ribosomal RNA analyses. Although most of our initial objectives for this project have been achieved, many questions regarding the origin and function of the tRNA arrays remain.

## Supplementary Material

Supplementary Figures

Supplementary Legends

Supplementary Table

Supplementary figures 1–5 and a supplementary table are available at Molecular Biology and Evolution online (http://www.mbe.oxfordjournals.org/).

## Figures and Tables

**Table 1:** Comparative Organization of tRNA Array Units in *Entamoeba* Species

tRNA Isoacceptor type	*Entamoeba dispar* SAW760^a^	*Entamoeba* *moshkovskii* FIC	*Entamoeba* *terrapinae* M	*Entamoeba invadens* IP-1
Ala^AGC^	[A^AGC^]	[A^AGC^]	[A^AGC^], [ADSS]	[A^AGC^]
Ala^TGC^	[ASD]	[ADSSD]	[ADSD], [ADRML]	[ASS]
Ala^CGC^	[ALL]	[ALL]	[ALLSL5]	[ALI]
Arg^ACG^	[R5]	[R5]	[IR]	[VMEDR5E]
Arg^CCG^	#	#	#	#
Arg^CCT^	[RT]	[RT]	[PTRL]	[PPTRL]
Arg^TCG^	[MR]	[MR]	[MRM5], [ADRML]	[MR]
Arg^TCT^	[R^TCT^]	[R^TCT^]	[R^TCT^]	[R^TCT^]
Asn^GTT^	[NK]	[NK]	[NK1], [NK2]	[NKQCK], [NK]
Asp^GTC^	[ASD], [SD]	[ADSSD]	[ADSD], [ADSS], [ADRML]	[VMEDR5E], [FVDTX]^b^, [EIDLLL]
Cys^GCA^	[SPPCK], [SQCK]	[SPPPCK], [SQCK]	[CK], [SQCK]	[NKQCK]
Gln^CTG^	[SQCK]	[SQCK]	[SQCK], [SQK]	[NKQCK]
Gln^TTG^	[TQ]	[TQ]	[VQ51], [VQ52]	[TQQ]
Glu^CTC^	[VME5]	[VME5]	[VME]	[VMEDR5E] [EIDLLL]
Glu^TTC^	[YE]	[YE]	[YE]	[VMEDR5E], [YE]
Gly^CCC^	#	#	#	#
Gly^GCC^	[G^GCC^]	[G^GCC^]	[G^GCC^]	[G^GCC^]
Gly^TCC^	[G^TCC^]	[G^TCC^]	[G^TCC^]	#, #SGI
His^GTG^	[H^GTG^]	[H^GTG^]	[H^GTG^]	[H^GTG^]
Ile^AAT^	[WI]	[WI1], [WI2]	[IR], [WI1], [WI2]	[WI], [EIDLLL]
Ile^TAT^	#	#	#	[ALI], #SGI
Leu^AAG^	[LT]	[LT], [PL]	[PTRL], [LT], [PL]	[PPTRL]
Leu^CAG^	[LS]	[LS]	[ALLSL5], [LLLSL5]	[LS]
Leu^CAA^	[ALL]	[ALL]	[ALLSL5], [LLLSL5]	[ALI], [EIDLLL]
Leu^TAA^	[ALL], #	[ALL], #	[ALLSL5], [ADRML], [LLLSL5]	[EIDLLL]
Leu^TAG^	#	#	#	#
Lys^CTT^	[NK]	[NK]	[NK1], [NK2]	[NKQCK], [NK]
Lys^TTT^	[SPPCK], [SQCK]	[SPPPCK], [SQCK]	[SQK], [CK], [SQCK]	[NKQCK]
eMet^CAT^	[MR]	[MR]	[MRM5], [ADRML]	[MR]
iMet^CAT^	[VME5]	[VME5], [MV5]	[VME]	[VMEDR5E]
Phe^GAA^	[VF]	[VF]	[VF]	[FVV5], [FVDTX]^b^
Pro^CGG^	[SPPCK]	[SPPPCK]	[SPPP]	[PPTRL]
Pro^AGG^	[SPPCK]	[SPPPCK]	[SPPP]	[SPP]
Pro^TGG^	[P^TGG^]	[SPPPCK]	[PTRL], [PL], [SPPP]	[SPP], [PPTRL]
Ser^AGA^	[SPPCK], [SQCK]	[SPPPCK], [SQCK]	[SQK], [SQCK], [SPPP]	[SPP]
Ser^GCT^	[ASD]	[ADSSD]	[ADSD], [ADSS]	[ASS]
Ser^CGA^	[LS]	[LS]	[ALLSL5], [LLLSL5]	[LS], #SGI
Ser^TGA^	[SD]	[ADSSD]	[ADSS]	[ASS]
Thr^AGT^	[LT]	[LT], [RT]	[PTRL], [LT]	[PPTRL]
Thr^CGT^	[TQ]	[TQ]	#	[TQQ]
Thr^TGT^	[TX]^b^	[TX]^b^	[TX]^b^	[FVDTX]^b^
Trp^CCA^	[WI]	[WI]	[WI1], [WI2]	[WI]
Tyr^GTA^	[YE]	[YE]	[YE]	[YE]
Val^CAC^	[VME5]	[VME5]	[VQ51], [VQ52]	[FVV5]
Val^GAC^	[VF]	[VF]	[VF]	[FVV5], [FVDTX]^b^
Val^TAC^	[V5]	[MV5]	[VME]	[VMEDR5E]

Note.—The tRNA gene content of each array is shown using the single-letter amino acid code. The 5S RNA gene is identified by “5” when present. Where 2 distinct variants of an array exist, the numbers 1 and 2 are appended to the gene complement. The *E. invadens* array containing only 5S RNA genes is not listed. # indicates dispersed tRNA genes. The dispersed cluster of 3 genes in *E. invadens* is indicated by #SGI.

a Unit organization in *Entamoeba histolytica* HM-1:IMSS (Clark, Ali, et al. 2006) and *E. dispar* SAW760 is identical except for [NK], which exists as 2 distinct arrays in *E. histolytica* but only 1 in *E. dispar*.

b X is a gene encoding the same unidentified small RNA in one array in each species (Banerjee and Lohia 2003; Clark, Ali, et al. 2006).

**Table 2:** Base Composition of Array Intergenic Regions

Organism	Mean A + T (%)	Range A + T (%)	Mean Pyrimidine (%)	Range Pyrimidine (%)
*Entamoeba histolytica* HM-1:IMSS	80.0	77.7–83.7	70.8	62.5–76.3
*Entamoeba dispar* SAW760	81.5	79.3–85.3	67.3	59.9–69.8
*Entamoeba moshkovskii* FIC	69.3	63.2–71.4	75.8	56.6–79.7
*Entamoeba terrapinae* M	71.2	67.6–74.3	53.2	50.4–56.8
*Entamoeba invadens* IP-1	81.8	66.4–84.7	56.9	51.0–74.7
