# Supplementary figures and images for: Patterns of Evolution in the Unique tRNA Gene Arrays of the Genus Entamoeba
Source: Mol Biol Evol. Author manuscript; Available in PMC 2009 Mar 9. (PMC2652664; doi:10.1093/molbev/msm238)

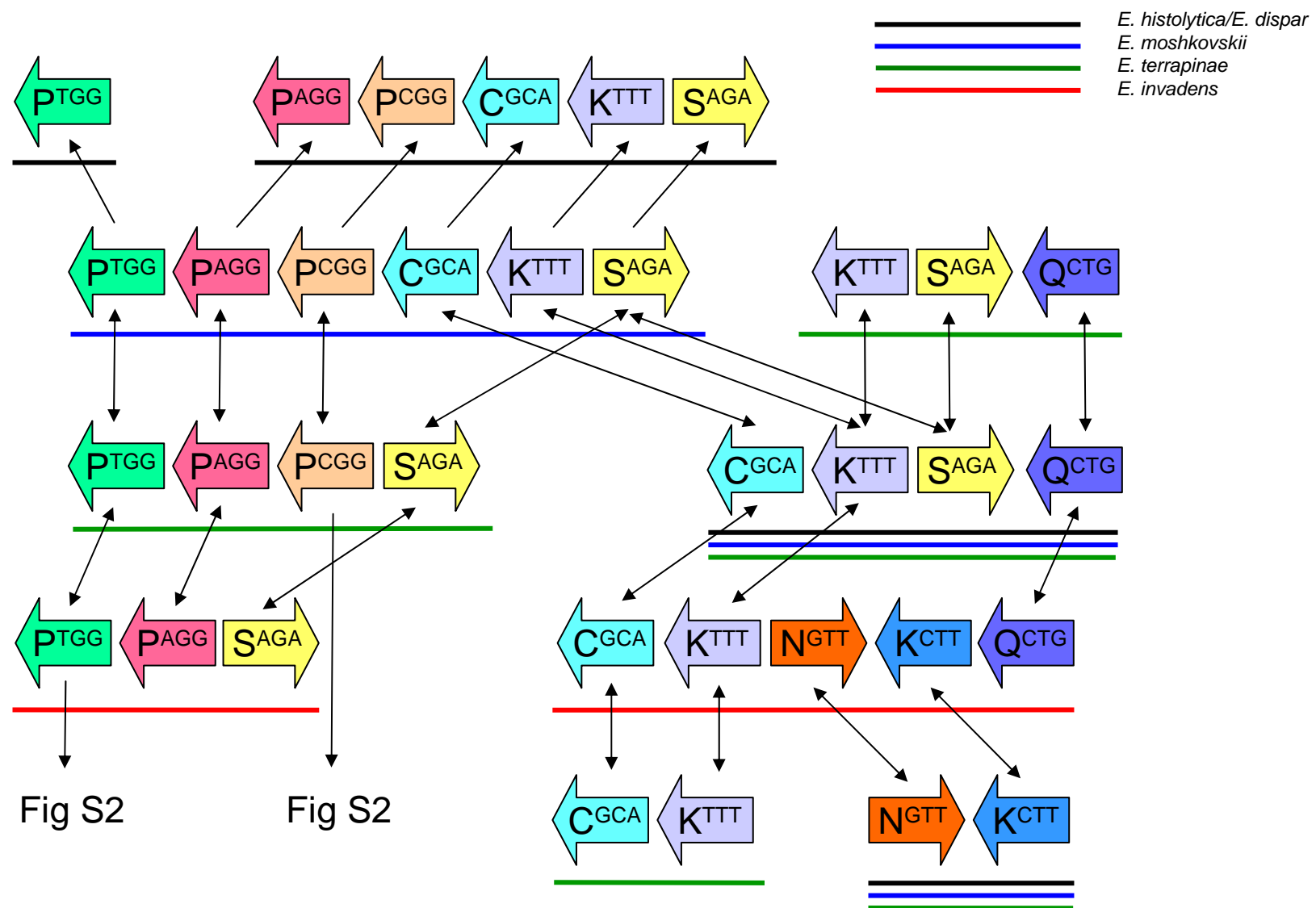

Figure S1

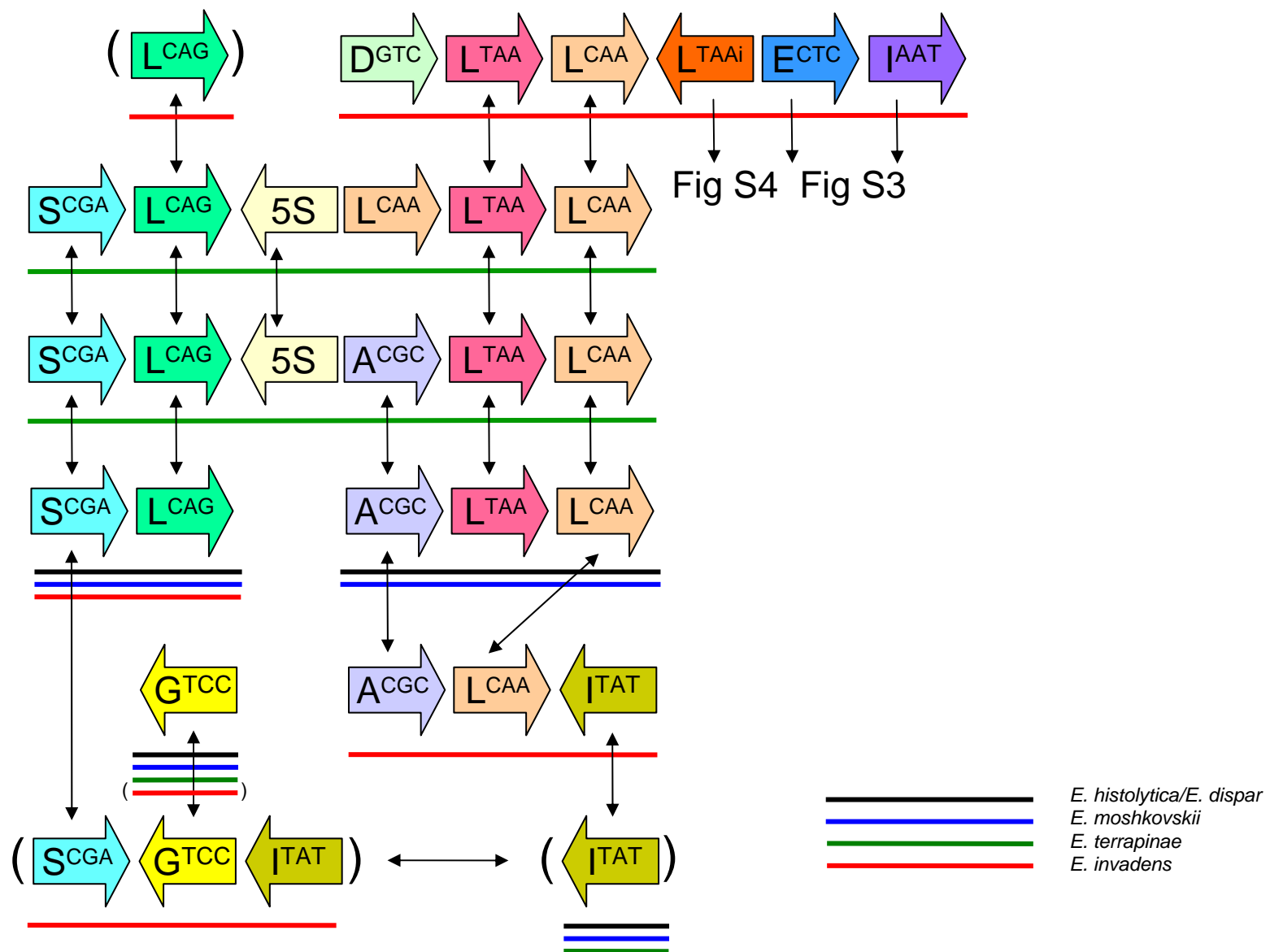

Figure S2

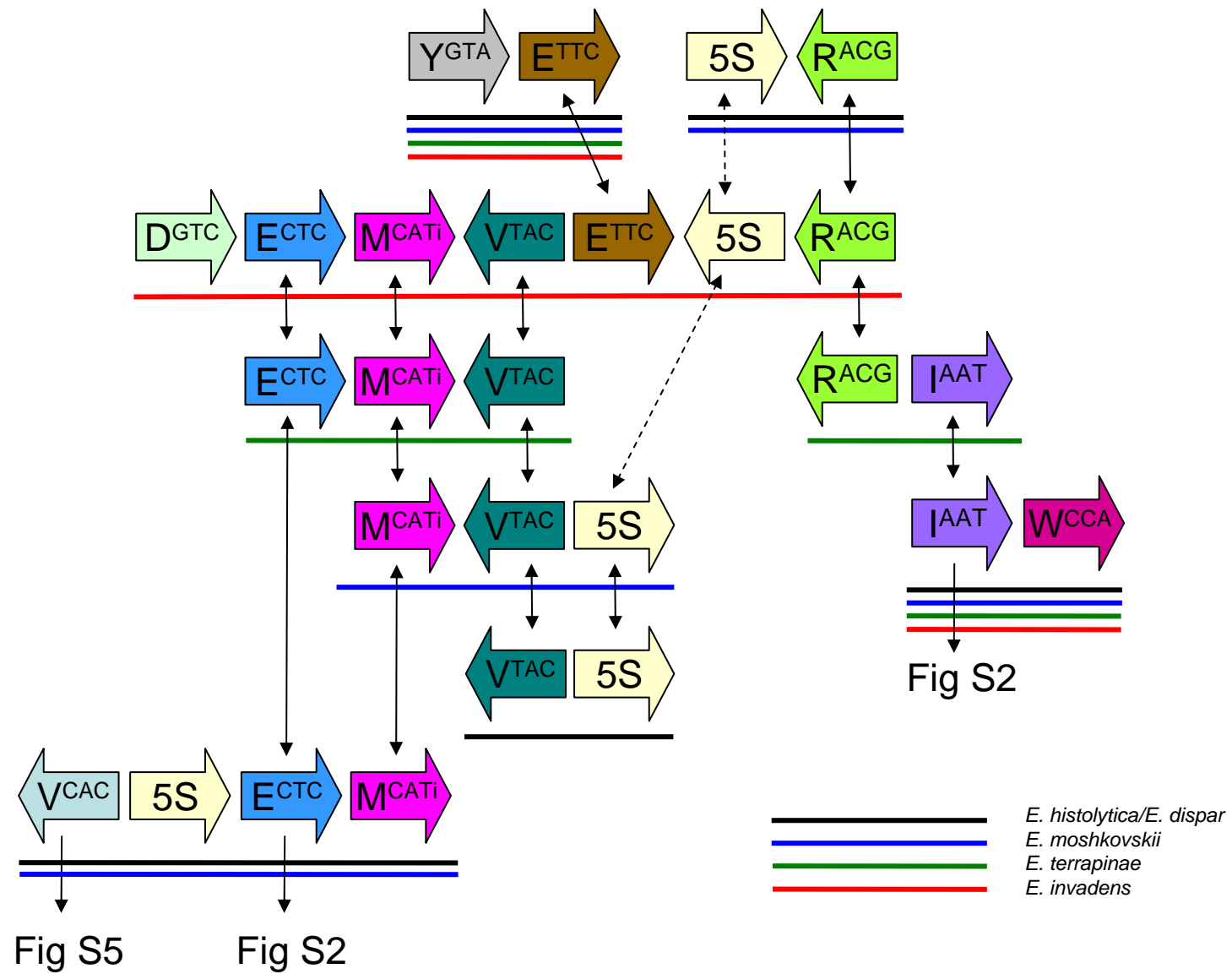

Figure S3

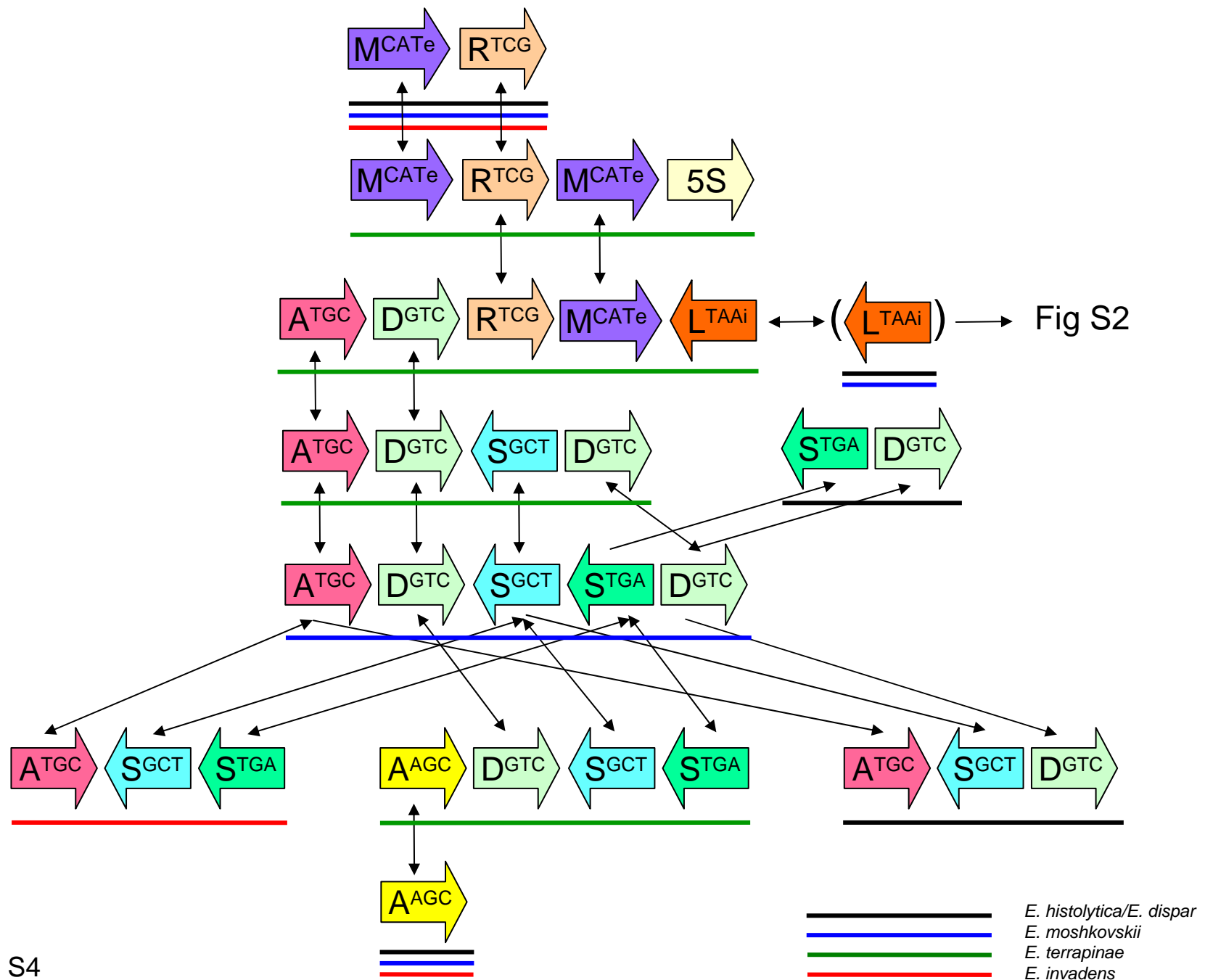

Figure S4

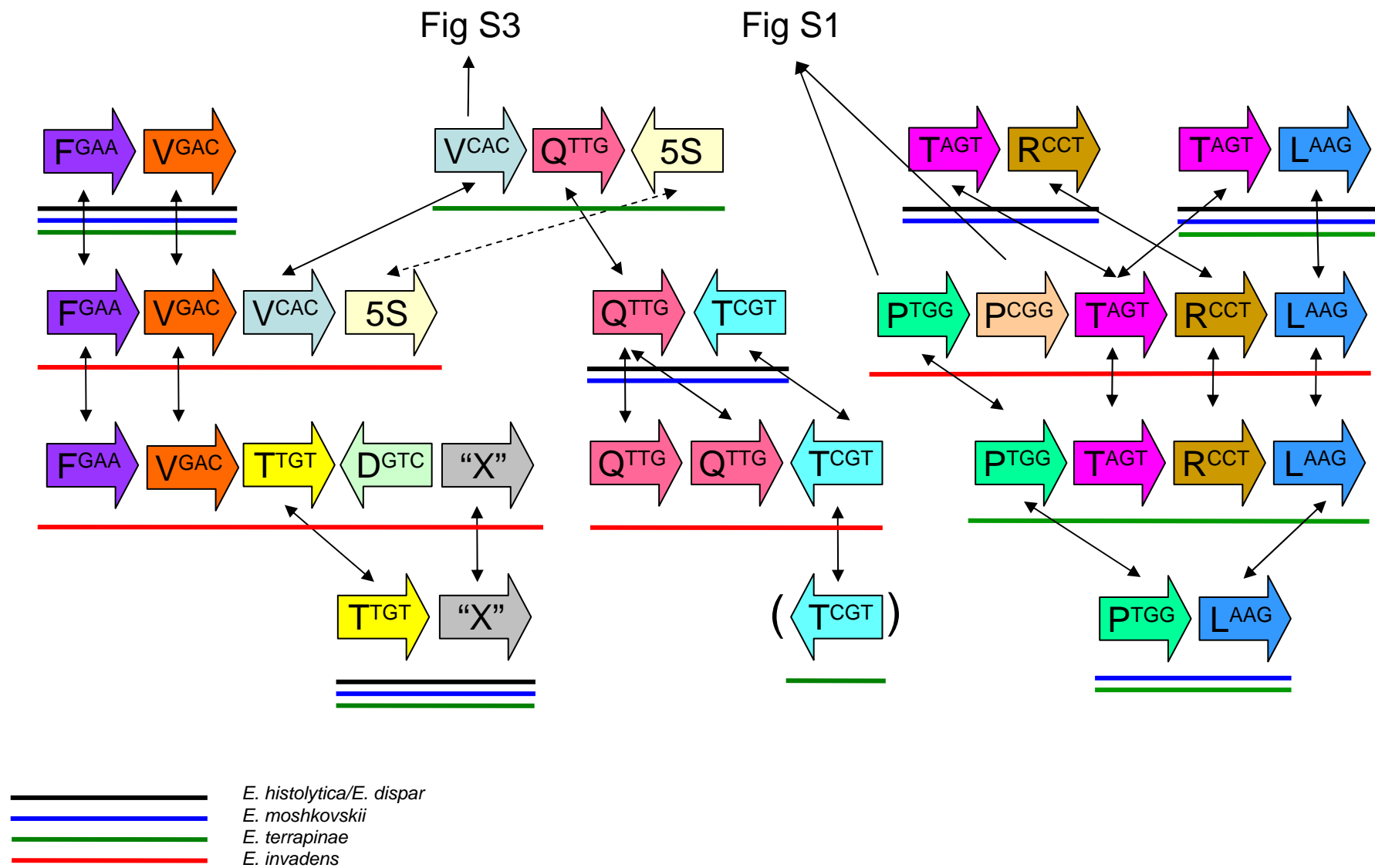

Figure S5

Supplement: Supplementary Figures [file NIHMS3038-supplement-suppfigs.pdf]
