# Supplementary material for: Patterns of Evolution in the Unique tRNA Gene Arrays of the Genus Entamoeba
Source: Mol Biol Evol. Author manuscript; Available in PMC 2009 Mar 9. (PMC2652664; doi:10.1093/molbev/msm238)
Supplement: Supplementary Legends [file NIHMS3038-supplement-supplegends.doc]

Supplementary file legends

Supplementary Figures S1-S5. Web of relationships among tRNA array unit organisations in the surveyed species (JPG files). The species sharing a particular unit organisation are indicated by the colored underlining. Colored arrows indicate the relative position and direction of the tRNA and 5S RNA genes. Where appropriate, dispersed genes are also shown (indicated in parentheses). Double headed arrows are used to indicate that the direction of change is not known in most cases. Dashed line arrows are used where the orientation of a gene has changed. Where the same gene is found in different figures the link to the other figure is indicated in both. Arrayed tRNA genes are shown in their unit contexts, although the order shown may not be identical to that in Table 1 in order to highlight the similarities. In figures S2 and L^TAAi^ indicates the version of this tRNA gene with an intron.

Supplementary Table 1. Accession numbers for sequences containing dispersed tRNA genes in the surveyed species.

Supplementary figures.pdf

Supplementary Table
