# Supplementary material for: Patterns of Evolution in the Unique tRNA Gene Arrays of the Genus Entamoeba
Source: Mol Biol Evol. Author manuscript; Available in PMC 2009 Mar 9. (PMC2652664; doi:10.1093/molbev/msm238)
Supplement: Supplementary Table [file NIHMS3038-supplement-supptable.doc]

Supplementary Table.

Accession numbers^a^ for sequences containing dispersed tRNA genes in the surveyed species.

|  | Species: |  |  |  |
| --- | --- | --- | --- | --- |
| tRNA gene: | *E. dispar* | *E. moshkovskii* | *E.terrapinae* | *E.invadens* |
|  |  |  |  |  |
| Arg^CCG^ | AANV02000073 | AM658904 | AM679532 | AM605407 |
|  |  |  | AM669811 | AM615364 |
|  |  |  |  |  |
| Gly^CCC^ | AANV02000059 | AM646700 | AM673411 | AM620263 |
|  | AANV02000099 | AM661067 |  | AM607836 |
|  | AANV02000339 | AM654499 |  | AM673411 |
|  | AANV02001288 | AM650478 |  |  |
|  | AANV02003057 | AM662310 |  |  |
|  |  |  |  |  |
| Gly^TCC^ |  |  |  | AM606324 |
|  |  |  |  | AM611344 |
|  |  |  |  | AM616772 |
|  |  |  |  | AM605344 |
|  |  |  |  |  |
| Cluster SGI |  |  |  | AM607739 |
| (see Table 1) |  |  |  | AM618187 |
|  |  |  |  |  |
| Ile^TAT^ | AM643248 | AM661421 | AM672640 |  |
|  | AM638140 | AM654591 | AM674806 |  |
|  | AM638137 | AM650263 | AM666782 |  |
|  | AM625481 | AM662221 | AM669732 |  |
|  | AM630428 | AM658584 | AM685351 |  |
|  | AM641719 |  |  |  |
|  | AM623864 |  |  |  |
|  |  |  |  |  |
| Leu^TAAi^ | AANV02000312 |  |  |  |
|  |  |  |  |  |
| Leu^TAG^ | AM637267 | AM663898 | AM680869 | AM614154 |
|  | AM627672 | AM651584 | AM686825 | AM619177 |
|  | AM623584 |  |  | AM604396 |
|  | AM628041 |  |  |  |
|  | AM641408 |  |  |  |
|  |  |  |  |  |
| Thr^CGT^ |  |  | AM669047 |  |
|  |  |  | AM672876 |  |
|  |  |  | AM682191 |  |

a - Entries containing these genes in *E. histolytica* are given in Ref 6. Accession numbers of the form AM****** are from sequences generated in this project. Accession numbers of the form AANV******** are given for genes not identified in this project but present in sequences from a separate project and deposited by TIGR.
